# Clinical and neuroimaging characteristics of Chinese dementia with Lewy bodies

**DOI:** 10.1371/journal.pone.0171802

**Published:** 2017-03-02

**Authors:** Shuai Liu, Xiao-Dan Wang, Ying Wang, Zhihong Shi, Li Cai, Shuling Liu, Tong Han, Yuying Zhou, Xinping Wang, Shuo Gao, Yong Ji

**Affiliations:** 1 Department of Neurology, Tianjin Huanhu Hospital, Tianjin, China; 2 Tianjin Dementia Institute, Tianjin Huanhu Hospital, Tianjin, China; 3 PET-CT Center, General Hospital of Tianjin Medical University, Tianjin, China; 4 Department of Radiology, Tianjin Huanhu Hospital, Tianjin, China; Banner Alzheimer's Institute, UNITED STATES

## Abstract

Dementia with Lewy bodies (DLB) is the second most common subtype of degenerative dementia. To our knowledge, available information about the clinical features of DLB in China remains limited. Our study therefore aimed to address this issue. Thirty-seven Chinese patients with probable DLB were recruited for this study. All subjects underwent neuropsychological assessment by trained neurologists, as well as undergoing MRI, ^11^C-PIB PET scans for Aβ deposition and ^18^F-FDG PET scans for regional cerebral glucose metabolism. Our results showed that the gender ratio of patients was 16:21 (F:M). The mean age of onset was 69.5 ± 9.0 years and the mean age at diagnosis was 71.8 ± 9.1 years. At diagnosis, the prevalence of three core clinical features of DLB was: 64.9% for fluctuating cognition, 73.0% for visual hallucinations and 62.2% for parkinsonism. The result from ^11^C-PiB PET and ^18^F-FDG PET scans confirmed Aβ deposition in the cortex and demonstrated hypometabolism in the bilateral temporoparietooccipital region, the frontal lobe, the insular lobe, and the posterior cingulate, precuneus and caudate nuclei. Our study elucidated the clinical features of Chinese DLB patients, and will improve the understanding and the early diagnosis of DLB in Chinese patients.

## Introduction

Dementia with Lewy bodies (DLB), which is defined pathologically as degeneration in the central, peripheral and autonomic nervous system associated with Lewy bodies (LBs) [1], was first proposed in 1996 (Perry R, 1996). As soon as the clinical and pathological guidelines were published in the same year, the clinical diagnosis of DLB became possible [2]. Thereafter, further clinical studies showed DLB to be the second most frequent form of dementia, after Alzheimer’s disease (AD) [3, 4]. The prevalence and incidence of DLB varies widely across reported studies, and the true prevalence and incidence may be largely underestimated since DLB has remained significantly under-diagnosed [5, 6]. A recent systematic review reported that the prevalence of DLB was 0.36% in the general population and 7.5% in clinical populations; while the incidence of DLB was 3.8% of new dementia cases and 0.87 cases per 1000 person-years [7].

The clinical manifestations of DLB are cognitive fluctuations, visual hallucinations and motor parkinsonism, but the condition may also manifest with rapid eye movement sleep behavior disorder and severe sensitivity to antipsychotic medications [8, 9]. Neuroimaging studies including structural imaging and functional imaging help make the diagnosis of DLB and distinguish it from other dementias [10]. A structural imaging study found a relative preservation of the hippocampal and medial temporal lobes in DLB [11]. Molecular and functional imaging studies showed a marked reduction of dopaminergic activity in the basal ganglia of DLB and hypometabolism in the occipital lobe with the temporal lobe relatively preserved [12–14]. The characteristic pathological lesions of DLB are Lewy bodies (LBs) and Lewy neurites, caused by the aggregation of α-synuclein [15]. However, AD pathology including amyloid-beta (Aβ) and tau has also been reported in DLB patients [16, 17].

Although first described several decades ago, DLB currently remains a diagnostic challenge due to its clinical and pathological heterogenicity and its similarity in certain respects with other neurodegenerative diseases. To date, aside from some case reports, there has been limited information available about the clinical features of DLB in China[18]. This present study aimed to improve the understanding of DLB in Chinese patients by analyzing the clinical features of 37 Chinese DLB patients.

## Methods

### Subjects

Thirty-seven DLB patients were recruited for this study from the cognitive disorder clinic at Tianjin Huanhu Hospital, Tianjin, China, between June 2011 and March 2015. To be included in the study, patients had to be diagnosed as probable DLB, according to the diagnostic criteria defined by McKeith in 2005 [6]. The determination of Parkinsonism was according to the diagnostic criteria defined by Calne [19], and the motor features and the severity were assessed with the Motor section of Unified Parkinson disease Rating Scale (UPDRS). To distinguish DLB from Parkinson’s disease associated with dementia, we excluded patients in whom cognitive impairment had occurred more than 1 year after they were diagnosed with the extrapyramidal syndrome. The 5 controls were healthy people without a family history of neurological or psychological disorders, who were the same controls we used in our previous study [20]. All subjects underwent clinical neuropsychological assessment by trained neurologists, as well as MRI, ^11^C-PIB PET scans for Aβ deposition and ^18^F-FDG PET scans for regional cerebral glucose metabolism. Patients’ information was collected including sex, age of onset, age of diagnosis, education, and clinical symptoms and signs. All patients underwent neuropsychological assessment including MMSE, MoCA, CDT, ADL and NPI. Some of the text of our previous study was reproduced in the present study [20].

### Ethics statement

Written informed consent was obtained from all subjects and/or their assigned surrogate decision-makers. The study was approved by the Tianjin Huanhu Hospital Ethics Committee.

### Magnetic resonance imaging

Magnetic resonance images were acquired using a 3.0T SIEMENS Tim Trio MRI scanner. A T1-weighted coronal image was acquired using a three-dimensional spoiled gradient recalled echo inversion recovery prepped sequence (repetition time [TR] = 11 ms, echo time [TE] = 4.94 ms, flip angle [FA] = 20°, 1 mm slice thickness [zero gap], 160 slices, field of view [FOV] = 230 mm × 230 mm). All of the images from the 3T were reconstructed to a size of 256 × 256 with an isotropic resolution of 1×1×1 mm.

### PET imaging

Head movement was minimized using a polyurethane immobilizer molded around the head. The PET images were acquired with a GE Discovery LS PET/CT scanner in the three-dimensional scanning mode, yielding 35 slices with 4.25 mm thickness that covered the entire brain. ^11^C-PIB PET scans were acquired during 90-min dynamic PET acquisition (34 frames: 4 × 15s, 8 × 30s, 9 × 60s, 2 × 180s, 8 × 300s, 3 × 600s). ^11^C-PIB was administered into an antecubital vein as a bolus injection, with a mean dose of 370–555 MBq. The images were reconstructed to a 128 × 128 matrix (2.5 × 2.5 mm^2^ pixel size).

The ^18^F-FDG study was conducted 1 h after the ^11^C-PIB PET scan using the same scanner, scanning mode, positioning and reconstruction matrix. The subjects received an intravenous injection of 250 MBq ^18^F-FDG and remained in a darkened, quiet room. A 10-min static PET emission scan was performed 60 min after the ^18^F-FDG injection.

### Quantification of ^11^C-PIB uptake

The uptake of ^11^C-PIB was quantified at the voxel level using the region-to-cerebellum ratio, which is identical to the standardized uptake value ratio (SUVR). This simplified quantification enables the utilization of a short 30-min image acquisition.

### Automated region-of-interest analysis

Standardized regions of interest (ROIs) were defined on the MRI template image representing brain anatomy, in accordance with the Montreal Neurological Institute (MNI) space. We merged and pooled subsets from the original Automated Anatomic Labeling (AAL) atlas to form the following ROIs: middle frontal gyrus (MFG), medial prefrontal cortex (MPFC), lateral temporal cortex (LTC), hippocampus and parahippocampus (HF+), inferior parietal lobe (IP), posterior cingulate cortex and precuneus (PCCPre), striatum, thalamus, occipital lobe (OL), superior temporal gyrus (STG), and supplementary motor area (SMA).

### ^11^C-PIB PET image analysis

The preprocessing of the ^11^C-PIB imaging data was performed using Statistical Parametric Mapping 8 (SPM8) software and MATLAB 2010b for Windows (Mathworks, Natick, MA, USA). First, ^11^C-PIB integral images (data corrected for radioactive decay summed from 60 to 90 min post-injection) were created from the dynamic PET images (frames 32 to 34) and coregistered to the subject’s MRI images. Second, the MRI images were segmented into three classes (gray matter, white matter, and cerebrospinal fluid) in SPM8 using 16 non-linear iterations and 7 × 9 × 7 basis functions. Third, the PET images and gray matter magnetic resonance images were normalized using a T1-weighted MRI template that was delivered with SPM to obtain normalization parameters. The application of a 0.5 threshold to the gray matter probability map created a gray matter probability map in the MNI space. The gray matter probability map was then coregistered to the AAL template, and the PET counts were extracted from the gray matter probability map and ROIs. The mean values for all of the regions were calculated from the integral ^11^C-PIB image. Target-to-cerebellum ratios were subsequently calculated for 11 bilateral regions.

### ^18^F-FDG PET image analysis

Spatial preprocessing and statistical analyses of ^18^F-FDG PET images were also performed in all of the subjects, using SPM8 software and MATLAB 2010b for Windows. We compared cerebral glucose metabolism in the DLB group with that of the control group. First, ^18^F-FDG PET images were converted to the ANALYZE format and then normalized to the MNI standard proportional stereotaxic space. Second, an isotropic 10 mm full-width half-maximum Gaussian spatial smoothing filter was applied to the image. Third, all of the comparisons of brain metabolism were performed on a voxel-by-voxel basis using a two-sample *t*-test. Statistical significance was determined using an extent threshold of 50 voxels. Regions that reached an uncorrected *P* value of less than 0.001 were considered statistically significant.

## Results

### Patient characteristics

Detailed patients’ characteristics are showed in Table 1. Thirty-seven DLB patients were included in this study (16 females and 21 males), with a mean onset age of 69.5 ± 9.0 years (range 50–89 years) and a mean age at diagnosis of 71.8 ± 9.1 years (range 52–89 years). The mean education duration was 10.3 ± 4.4 years. The neuropsychological test scores were: 16.6 ± 7.4 for MMSE, 1.8 ± 1.5 for CDT, 40.4 ±17.4 for ADL and 9.6 ± 7.0 for MoCA, 13.9 ± 12.4 for UPDRS, respectively. Of the tested parameters, attention, calculation, delayed recall, executive function and visuospatial ability were most affected.

**Table 1:** The characteristics of Chinese DLB patients.

Patients’ characteristics	
**Sex (F/M)**	16/21
**Age of onset (years)**	69.5±9.0
**Age of diagnosis (years)**	71.8±9.1
**Education (years)**	10.3±4.4
**MMSE**	16.6±7.4
**CDT**	1.8±1.5
**ADL**	40.4±17.4
**MoCA**	9.6±7.0
**UPDRS**	13.9±12.4

Data are the mean ± SD (except gender).

### Clinical manifestations

All patients’ symptoms of onset were recorded. At the onset of the disease, 48.6% of patients showed cognitive impairment as the initial symptom, 18.9% of patients showed visual hallucinations, 10.8% of patients showed cognitive impairment and parkinsonism, 8.1% of patients showed psychiatric symptoms (excepting visual hallucinations), 5.4% of patients showed cognitive impairment and visual hallucinations, 2.7% of patients showed parkinsonism, 2.7% of patients showed cognitive impairment and psychiatric symptoms (excepting visual hallucinations), and 2.7% of patients showed blurred vision (Table 2).

**Table 2:** First symptom at onset of Chinese DLB patients.

Symptoms	Percentage
Cognitive impairment	48.6%
Visual hallucinations	18.9%
Cognitive impairment and parkinsonism	10.8%
Psychiatric symptoms (except visual hallucinations)	8.1%
Cognitive impairment and visual hallucinations	5.4%
Parkinsonism	2.7%
Cognitive impairment and psychiatric symptoms (except visual hallucinations)	2.7%
Blurred vision	2.7%

**Fig 1:**
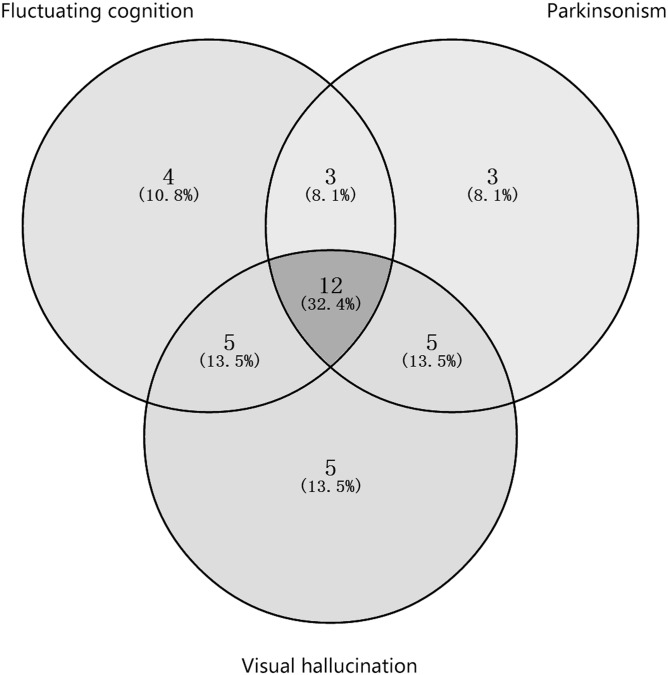
The number and percentage of cases shared specific core features within the data set at the diagnosis.

At the time of diagnosis of the disease, the core features of the Chinese DLB patients were recorded (Table 3). Of the 37 patients, 64.9% displayed fluctuating cognition. Most (83.8%) reported memory impairment, while 59.4% of patients showed visuospatial dysfunction, 24.3% of patients showed language dysfunction, 18.9% of patients showed executive dysfunction, and 5.4% of patients showed declined attention. In addition, 62.2% of the patients showed symptoms of Parkinsonism including rigidity (35.1%), postural instability and gait difficulty (29.7%), bradykinesia (21.6%), and rest tremor (18.9%). Furthermore, 73.0% of the patients manifested visual hallucinations which were recurrent, well-formed and detailed. The number and percentage of cases shared specific core features within the data set at the diagnosis were showed in Fig 1. Four patients (10.8%) displayed only fluctuating cognition, three patients (8.1%) displayed only Parkinsonism, five patients displayed (13.5%) only visual hallucinations, three patients (8.1%) displayed fluctuating cognition and Parkinsonism, five patients (13.5%) displayed fluctuating cognition and visual hallucinations, five patients (13.5%) displayed Parkinsonism and visual hallucinations, and twelve patients (32.4%) displayed all three core features.

**Table 3:** The core symptoms of Chinese DLB patients.

Symptoms	Percentage
cognitive impairment	
Fluctuating cognition	64.9%
Memory impairment	83.8%
Visuospatial dysfunction	59.4%
Language dysfunction	24.3%
Executive dysfunction	18.9%
Declined attention	5.4%
Parkinsonism	62.2%
Rigidity	35.1%
Postural instability and gait difficulty	29.7%
Bradykinesia	21.6%
Rest tremor	18.9%
Visual hallucinations	73.0%

In addition we analyzed the percentages of the suggestive features and the supportive features occurring in the Chinese DLB patients (Table 4). We found that 21.6% had the suggestive feature of REM sleep behavior disorder and 21.1% of the 19 patients who were treated with neuroleptics had suggestive feature of severe neuroleptic sensitivity. As for the supportive features, 75.7% of the patients showed psychiatric symptoms other than visual hallucinations, including irritability (54.1%), depression (43.2%), anxiety (40.5%), apathy (37.8%), delusions (35.1%), compulsion (24.3%), derepression (8.1%), auditory hallucinations (10.8%) and euphoria (5.4%). Moreover, 32.4% of the patients displayed autonomic dysfunction, including constipation (21.6%), syncope (18.9%), orthostatic hypotension (8.1%), frequency of urination and urgency of urination (5.4%), urinary retention (5.4%), hidrosis (5.4%) and hydrostomia (2.7%). The percentage of cases of transient, unexplained loss of consciousness was the same as that of repeated falls, at the figure of 35.1%.

**Table 4:** The suggestive and the supportive features of Chinese DLB patients.

Features	Percentage	Number/Total
**REM sleep behavior disorder**	21.6%	8/37
**Severe neuroleptic sensitivity**	21.1%	4/19
**Psychiatric symptoms**	75.7%	28/37
Irritability	54.1%	20/37
Depression	43.2%	16/37
Anxiety	40.5%	15/37
Apathy	37.8%	14/37
Delusion	35.1%	13/37
Compulsion	24.3%	9/37
Derepression	8.1%	3/37
Auditory hallucination	10.8%	4/37
Euphoria	5.4%	2/37
**Autonomic dysfunction**	32.4%	12/37
Constipation	21.6%	8/37
Syncope	18.9%	7/37
Orthostatic hypotension	8.1%	3/37
Frequency and urgency of urination	5.4%	2/37
Urinary retention	5.4%	2/37
Hidrosis	5.4%	2/37
Hydrostomia	2.7%	1/37
**Transient, unexplained loss of consciousness**	35.1%	13/37
**Repeated falls**	35.1%	13/37

### Aβ deposition analysis with ^11^C-PiB PET and cerebral glucose metabolism analysis with ^18^F-FDG PET

**Fig 2:**
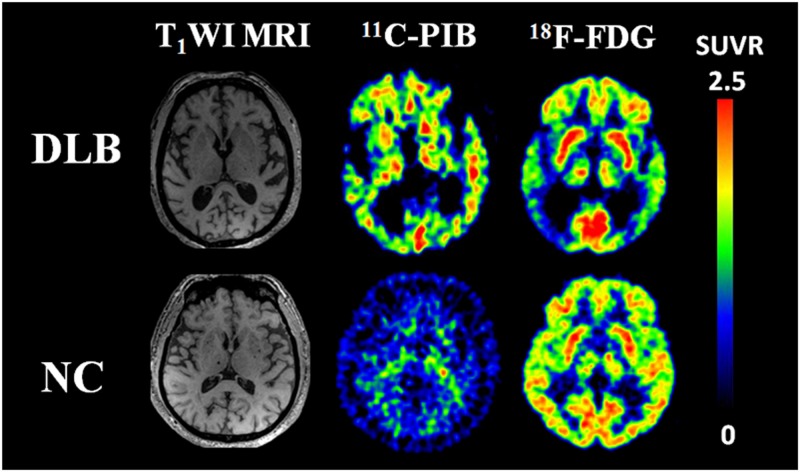
MRI (left), PiB (middle) and FDG (right) images of a Chinese DLB patient and a healthy control. PiB and FDG images are quantified by SUVR with the displayed color scales. In DLB patient, the clearance rate of radioactivity was slower symmetrically or asymmetrically in the cortex of the frontal lobe, parietal lobe, lateral temporal lobe, precuneus, posterior cingulate and occipital lobe.

**Fig 3:**
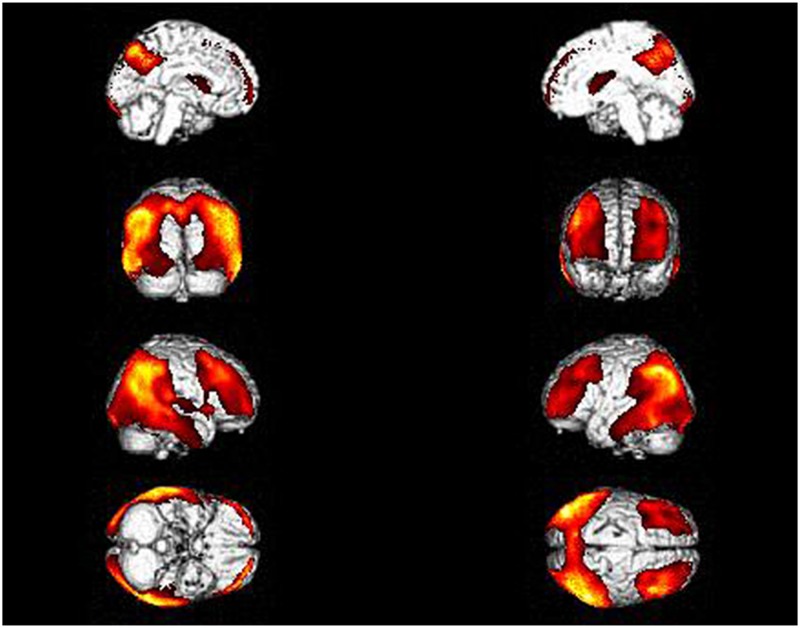
Topography of hypometabolism in Chinese DLB patients. (See details in Methods and Results).

We analyzed Aβ deposition in brain with 11C-PiB PET in the Chinese DLB patients. All of the patients were Aβ positive. After 45 min of PIB injection, visual analysis showed that the clearance rate of radioactivity was slower symmetrically or asymmetrically in the cortex of the frontal lobe, parietal lobe, lateral temporal lobe, precuneus, posterior cingulate and occipital lobe (Fig 2). The SUVR of DLB was significantly higher than in controls in IP, LTC, MFG, MPFC, PCCPre, OL, SMA, STG, and striatum (S1 Table). In the same patient cohort, we also analyzed cerebral glucose metabolism with ^18^F-FDG PET, which showed hypometabolism in the bilateral temporoparietooccipital region, frontal lobe, insular lobe, posterior cingulate, precuneus and caudate nuclei (Fig 2). SPM analyses revealed significant hypometabolism in the left middle temporal gyrus (BA39, 21), inferior temporal gyrus (BA37, 20), superior occipital gyrus (BA19), angular gyrus (BA39), precuneus (BA7), and supramarginal gyrus (BA40), and the right middle frontal gyrus (BA8), precentral gyrus (BA9), superior temporal gyrus (BA39), middle temporal gyrus (BA37, 39), inferior temporal gyrus (BA20), inferior parietal lobule (BA40), superior occipital gyrus (BA19), precuneus (BA7,19) and supramarginal gyrus (BA40) (Fig 3 and S2 Table).

## Discussion

The present study is the first to explore, in a single clinic center, the clinical features of Chinese DLB patients, as well as the pattern of cerebral Aβ deposition and glucose metabolism in these patients. Our results showed that amongst these patients, the gender ratio was 16:21 (F:M), the mean onset age was 69.5 ± 9.0 years and the mean diagnosed age was 71.8 ± 9.1 years. Furthermore, the results of neuropsychological tests indicated that both the cognitive functioning and the daily life capacity of DLB patients had declined by the time they visited our clinic. The disease usually initially presents with core clinical features; the percentage of patients with the three individual core clinical features at time of diagnosis was 64.9% for fluctuating cognition, 73.0% for visual hallucinations and 62.2% for parkinsonism. The result from ^11^C-PiB PET and ^18^F-FDG PET scans confirmed Aβ deposition and revealed hypometabolism in the cortex. Our study provides a better understanding of DLB in Chinese patients and will help to improve the identification and the early diagnosis of patients with this condition.

In the present study, we found a slight preponderance of males in our Chinese DLB patients. However, past studies of gender differences in DLB patients have shown inconsistent results [7]. Some researchers have reported disproportionately more females with the disease, while others have reported disproportionately more males [21, 22]. The condition is thought to first occur in the later years of life, generally at ages 60 to 90 [2, 23], with a peak incidence reported in the 7th decade of life [24]. In our study the age of onset of the condition was around the 7th decade of life, which is consistent with previous reports.

Our study confirmed that cognitive decline is the most common symptom at the onset of the disease, with visual hallucinations and parkinsonism less likely to be manifest [18]. However, importantly, DLB can also begin with psychiatric symptoms other than visual hallucinations [8, 25]. Although a previous study has reported that fluctuating cognition occurred in 80% or more of individuals with DLB [26], we found a slightly lower frequency of fluctuating cognition was evident in the Chinese DLB patients in our study. We believe our result was probably a more accurate reflection of the real prevalence of fluctuating cognition in Chinese DLB patients. Moreover, our study showed that memory deficits is the most common component of cognitive impairment in Chinese DLB patients. The results from MMSE, MoCA and CDT tests indicated that, besides memory, visuospatial function, language, executive function and attention were also affected. Previous studies have suggested that in the early stages of the disease, DLB patients tend to exhibit pronounced visual-perceptual, attentional and frontal executive impairments, whereas memory impairment may not necessarily be evident [6, 27–29]. Nevertheless in our study, most of the patients complained of memory decline when first affected by the disorder. This phenomenon might have resulted from the small size of the sample or it may represent an unique characteristic feature in Chinese DLB, which needs to be further studied with a bigger sample size. In our study, the frequency of Parkinsonism in DLB, another core clinical feature of the condition, was also consistent with that reported in the literature [30, 31]. The features of parkinsonism in DLB, which may include tremor, rigidity, bradykinesia, diminishing facial expressions and impaired fine motor performance, may differ from those found in idiopathic PD [32]. In line with previous studies, which have reported the prevalence rate of visual hallucinations to be in excess of 60% [33], we found in our study that 73.0% of Chinese DLB patients showed visual hallucinations. These seem to be associated with the impairment of anterior and posterior regions (secondary visual areas, orbitofrontal cortex and anterior cingulate cortex) involved in a top-down (impaired attentional binding) and a bottom-up (perceptual processes) mechanism, respectively.

Patients were also investigated for the presence of suggestive features of Chinese DLB. We found that neither REM sleep behavior disorder nor severe neuroleptic sensitivity were common in Chinese DLB patients. In our study, the percentage of those patients with the suggestive features was lower than in the previous study [25]. It has been reported that at least 80% of patients with DLB experience some form of neuropsychiatric symptoms [33]. Our data produced results similar to the previous study. Moreover, in addition to visual hallucinations, patients with DLB can also experience other psychiatric symptoms such as irritability, depression, anxiety, apathy, delusion, compulsion, derepression, auditory hallucination and euphoria, as was demonstrated in our study. In addition, a small number of patients also showed supportive features including autonomic dysfunction, transient, unexplained loss of consciousness and repeated falls. It has been reported that in DLB some noncognitive symptoms of the condition such as constipation, hyposmia and postural dizziness can predate the onset of memory impairment by years [34]. Therefore, the presence of these suggestive and supportive features may help to improve the recognition of DLB in clinical practice.

Although the main pathologic feature of DLB is the Lewy bodies and Lewy neurites, most patients with DLB also have the pathology typically seen in AD patients [35, 36]. Using ^11^C-PiB PET, we analyzed amyloid-beta deposition in the Chinese DLB patients. The results confirmed the presence of the deposition of Aβ in the cortex of the Chinese DLB patients, which is similar to that seen in AD using visual analysis. Indeed, the deposition of Aβ into neuritic and diffuse plaques is present in approximately 85% of cases of DLB [37]. Some studies have reported similarities in Aβ deposition in DLB and AD cases, while other studies have reported lower mean cortical Aβ ligand binding in DLB patients [38]. Some studies have established a distinctive pattern of hypometabolism in the occipital cortex and visual association cortices [37, 39, 40]. However, though the finding is not consistent with previous studies, the data from ^18^F-FDG PET indicated hypometabolism in the bilateral temporoparietooccipital region, frontal lobe, insular lobe, posterior cingulate, precuneus and caudate nuclei.

## Conclusions

In conclusion, our study identified and elucidated the clinical features of Chinese DLB patients. Our results indicated that DLB in the Chinese population is a late-onset dementia with a slight male preponderance. Cognitive impairment is the most common symptom at the onset of the disease and memory deficits are the most common component of cognitive impairment in Chinese DLB patients. The prevalence of three core clinical features at diagnosis was 64.9% for fluctuating cognition, 73.0% for visual hallucinations and 62.0% for parkinsonism. The result from ^11^C-PiB PET and ^18^F-FDG PET scans confirmed Aβ deposition and hypometabolism in the cortex. Our findings provide a comprehensive view of DLB in Chinese patients.

## Supporting information

S1 Table The PIB standardized uptake value ratio in Regions Of Interest (ROIs) of controls and DLB patients. (DOCX)

S2 Table The SPM values for FDG of DLB patients vs. Controls. (DOCX)
