# Supplementary material for: Clinical and neuroimaging characteristics of Chinese dementia with Lewy bodies
Source: PLoS One. 2017 Mar 2;12(3):e0171802. doi: 10.1371/journal.pone.0171802 (PMC5333817; doi:10.1371/journal.pone.0171802)
Supplement: S1 Table — (DOCX) [file pone.0171802.s001.docx]

**S1 Table. The PIB standardized uptake value ratio in regions of interest (ROIs) of Controls and DLB patients.**

| **ROIs** | **Control** | **DLB** |
| --- | --- | --- |
| **HF+** | 1.0 ± 0.1 | 1.3 ± 0.1 |
| **IP** | 1.1 ± 0.1 | 1.8 ± 0.2* |
| **LTC** | 1.2 ± 0.2 | 1.9 ± 0.2* |
| **MFG** | 1.1 ± 0.04 | 1.8 ± 0.1* |
| **MPFC** | 1.1 ± 0.1 | 1.9 ± 0.2* |
| **PCCPre** | 1.2 ± 0.1 | 1.9 ± 0.3* |
| **OL** | 1.2 ± 0.1 | 2.0 ± 0.3* |
| **SMA** | 1.1 ± 0.1 | 1.8 ± 0.2* |
| **STG** | 1.1 ± 0.1 | 1.6 ± 0.1* |
| **Striatum** | 1.1 ± 0.1 | 1.7 ± 0.1* |
| **Thalamus** | 1.2 ± 0.1 | 1.3 ± 0.1 |

Data are the mean ± SD. HF+:hippocampus and parahippocampus, IP: inferior parietal lobe, LTC: lateral temporal cortex, MFG: middle frontal gyrus, MPFC: medial prefrontal cortex, PCCPre: posterior cingulate cortex and precuneus, OL: occipital lobe, SMA: supplementary motor area, STG: superior temporal gyrus. *p<0.05 vs. control (Two-way ANOVA followed by Bonferroni’s *post-hoc* test).
