# Supplementary material for: Clinical and neuroimaging characteristics of Chinese dementia with Lewy bodies
Source: PLoS One. 2017 Mar 2;12(3):e0171802. doi: 10.1371/journal.pone.0171802 (PMC5333817; doi:10.1371/journal.pone.0171802)
Supplement: S2 Table — (DOCX) [file pone.0171802.s002.docx]

**S2 Table. The** **SPM values for FDG of DLB patients vs. Controls.**

| **MNI Coordinates** | | | **Cerebral Gyrus** | **BA** | **T** | **Z** |
| --- | --- | --- | --- | --- | --- | --- |
| **x** | **y** | **z** |  |  |  |  |
| -64 | -42 | -4 | L Middle Temporal Gyrus | BA 21 | 9.79 | 5.69 |
| -62 | -46 | -2 | L Middle Temporal Gyrus | BA 21 | 10.30 | 5.83 |
| -60 | -50 | -0 | L Middle Temporal Gyrus | BA 21 | 10.17 | 5.79 |
| -58 | -54 | -2 | L Middle Temporal Gyrus | BA 21 | 9.98 | 5.74 |
| -50 | -70 | 16 | L Middle Temporal Gyrus | BA 39 | 11.19 | 6.04 |
| -48 | -72 | 20 | L Middle Temporal Gyrus | BA 39 | 11.04 | 6.01 |
| -40 | -80 | 20 | L Middle Temporal Gyrus | BA 39 | 15.64 | 6.87 |
| -62 | -22 | -24 | L Inferior Temporal Gyrus | BA 20 | 9.08 | 5.50 |
| -60 | -46 | -18 | L Inferior Temporal Gyrus | BA37 | 9.74 | 5.68 |
| -36 | -78 | 34 | L Superior Occipital Gyrus | BA 19 | 13.60 | 6.53 |
| -58 | -54 | 30 | L Supramarginal Gyrus | BA 40 | 11.89 | 6.20 |
| -14 | -68 | 52 | L Precuneus | BA 7 | 13.33 | 6.48 |
| -4 | -62 | 44 | L Precuneus | BA 7 | 10.32 | 5.83 |
| -46 | -62 | 44 | L Angular Gyrus | BA 39 | 13.18 | 6.45 |
| 38 | 30 | 44 | R Middle Frontal Gyrus | BA 8 | 11.31 | 6.07 |
| 48 | 26 | 36 | R Precentral Gyrus | BA 9 | 10.19 | 5.80 |
| 50 | 26 | 32 | R Precentral Gyrus | BA 9 | 10.48 | 5.87 |
| 58 | -54 | 24 | R Superior Temporal Gyrus | BA 39 | 12.15 | 6.25 |
| 50 | 20 | 42 | R Middle Frontal Gyrus | BA 8 | 9.69 | 5.67 |
| 52 | -68 | 20 | R Middle Temporal Gyrus | BA 39 | 10.98 | 5.99 |
| 54 | -66 | 8 | R Middle Temporal Gyrus | BA 37 | 8.91 | 5.45 |
| 66 | -46 | -10 | R Middle Temporal Gyrus | BA 37 | 9.89 | 5.72 |
| 68 | -26 | -22 | R Inferior Temporal Gyrus | BA 20 | 11.64 | 6.14 |
| 54 | -48 | 44 | R Inferior Parietal Lobule | BA 40 | 11.77 | 6.17 |
| 56 | -42 | 44 | R Inferior Parietal Lobule | BA 40 | 11.30 | 6.07 |
| 42 | -74 | 26 | R Superior Occipital Gyrus | BA 19 | 11.32 | 6.07 |
| 56 | -54 | 38 | R Supramarginal Gyrus | BA 40 | 12.72 | 6.36 |
| 6 | -54 | 38 | R Precuneus | BA 7 | 12.39 | 6.30 |
| 38 | -64 | 46 | R Precuneus | BA 19 | 12.26 | 6.27 |

MNI: Montreal Neurological Institute L: Left, R: Right, BA: Brodmann’s area.
